# A Review on Ethno-Medicinal and Pharmacological Activities of *Sphenocentrum jollyanum* Pierre

**DOI:** 10.3390/medicines4030050

**Published:** 2017-07-03

**Authors:** Olubukola Sinbad Olorunnisola, Olumide Samuel Fadahunsi, Peter Adegbola

**Affiliations:** Department of Biochemistry, Faculty of Basic Medical Sciences, Ladoke Akintola University of Technology, Ogbomoso, PMB 4000, Oyo State, Nigeria; osolorunnisola@lautech.edu.ng (O.S.O.); useablevesselofgod@gmail.com (P.A.)

**Keywords:** ethno-medicinal uses, pharmacological activities, phytochemical profile, *Sphenocentrum jollyanum* Pierre

## Abstract

*Sphenocentrum jollyanum* Pierre is a member of a diverse family of plants known as Menispermaceae. They are famous for a plethora of important biological functions. *S. jollyanum* is a shrub native to the tropical forest zones of West Africa and thrives in deep shade. It is widely cultivated in Cameroun, Sierra Leone, Nigeria, Ghana, and Côte d’Ivoire. *S. jollyanum* is employed in folk medicine as a cure for wounds, fever, coughs, high blood pressure, breast tumor, constipation, and as an aphrodisiac. Phytochemical investigations revealed that the plant is a rich source of secondary metabolites such as annin, alkaloids, saponins, and flavonoids. Pharmacological activities include anti-diabetic, anti-inflammatory, anti-bacterial, anti-viral, anti-malarial, angiogenic, and anxiogenic. Thus, this present review summarizes the phytochemical and nutritional constituents and important biological studies on various crude extracts, fractions, and isolated principles of all morphological organs of *S. jollyanum*.

## 1. Introduction

Globally, the employment of medicinal plants as a substitute for orthodox drugs in the management of various diseases has been increasing due to the unavailability of modern health facilities, relative availability of medicinal herbs, poverty, and recent revelations that they possess active compounds that may be responsible for different biological and pharmacological actions [1]. According to Newman and Cragg [2], natural materials are the source of about two -third of all drugs developed in the past three decades. According to a World Health Organization (WHO) report, it is estimated that close to 80% of the people living in the third world nations of the world depend on traditional and complementary medicines for their basic health care [3]. Thus, this review is aimed at elucidating the traditional and biological importance of *Sphenocentrum jollyanum*.

*S. jollyanum* Pierre is a member of the plant family Menispermaceae (Table 1). These are diverse group of plants which are popular for important biological activities. *S. jollyanum* is a shrub native to the tropical forest zones of West Africa and is widely distributed in Sierra Leone, Nigeria, Ghana, Ivory Coast, and Cameroun [4]. It is extensively employed in folkloric medicine in the management of various ailments. *S. jollyanum* is commonly known locally as “*Aduro kokoo*” (red medicine), “*Okramankote*” (dog’s penis), and “*Krakoo*” among the Akan and Asante tribes of Ghana. In South-Western Nigeria, the plant is traditionally known as Akerejupon or Ajo, Oban abe in Republic of Benin, and Ouse-abe in Côte d’Ivoire [5].

## 2. Plant Description

**Figure 1:**
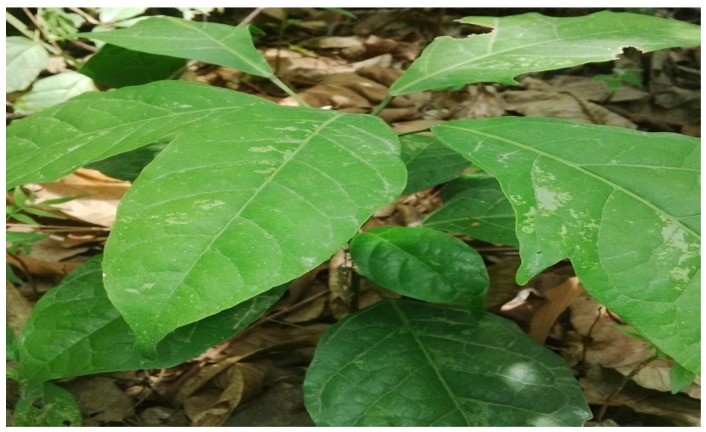
*Sphenocentrum jollyanum* in its natural habitat.

**Figure 2:**
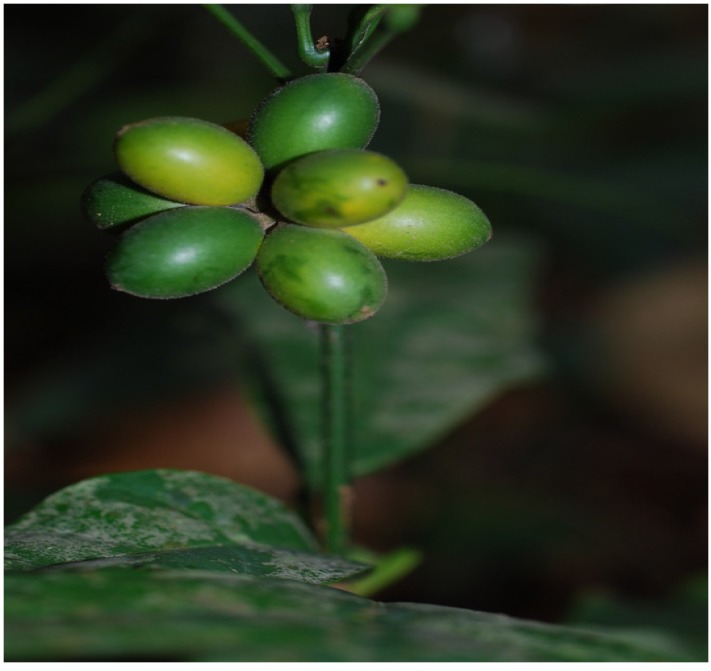
*Sphenocentrum jollyanum* fruits.

*Sphenocentrum jollyanum* (Figure 1) is a perennial under growth of dense forest, which thrives in deep shade, from sea-level up to 400 m altitude. It is mostly found in regions withmean annual rainfall of 1800 mm or more, mean minimum temperature of 20 °C, and mean maximum of 29 °C. It grows to an average height of about 1.5 m, with few scanty branches. *S. jollyanum* leaves are wedge-shaped, about 5–12 cm wide, smooth onboth sides, and can grow up to about 20 cm long with a small-arrowed apex [6,7]. The fruit (Figure 2) occurs as clusters, ovoid-ellipsoid, with a single large oval-shaped seed. It is fleshy, edible, and bright yellow or orange when ripe [8,9]. The roots are visibly bright yellow and are characterized by a sour acidic taste that causes things eaten thereafter to taste sweet [8].

## 3. Ethno-Medicinal Uses

Different parts of *S. jollyanum* are traditionally employed in folkloric medicine. In Ghana, the root of the plant is widely used by the men as an aphrodisiac. It is steeped in alcohol for few days to allow extraction of some constituents, which is thereafter drank as bitters to strengthen penile erection, and this effect is known to be long-lasting [10,11].

It has been documented in several literature studies that the root is employed for its effectiveness in stimulating the central nervous system (CNS), the management of mental and inflammatory disorders, pain and depression [12]. The dried, powdered root is used in combination with some anti-malarial plant as panacea for fever and muscular pains. Aerial parts of the plant (leafy twigs and fruits) are vastly employed in the treatment of chronic wounds, feverish conditions, and coughs when combined with *Piper guineense* (West African pepper) and lime juice [8,9].

In Nigeria, the roots are chewed in traditional medicine to relieve constipation, promote appetite, and increase digestion. All morphological organs of *S. jollyanum* are important ingredients in the management of sickle cell disease (SCD) [8]. Traditional healers in Ghana and Côte d’Ivoire have also reported the roots to possess medicinal properties against high blood pressure, breast tumor, irregular menstrual cycle, and diabetes mellitus [7,13,14,15,16]. Furthermore, the powdered roots are eaten in combination with *Aframomum melegueta* (alligator pepper) seeds, salt, and *Elaeis guineensis* (African oil palm) in the management of abdominal discomfort [17]. The charred fruits are used in the treatment of fibroids and as edible anti-fatigue snack [9,18]. It is also reported that the leaves decoctions are used in expelling intestinal parasites and to stop spitting of blood [15].

## 4. Phytochemical and Proximate Analysis 

**Figure 3:**
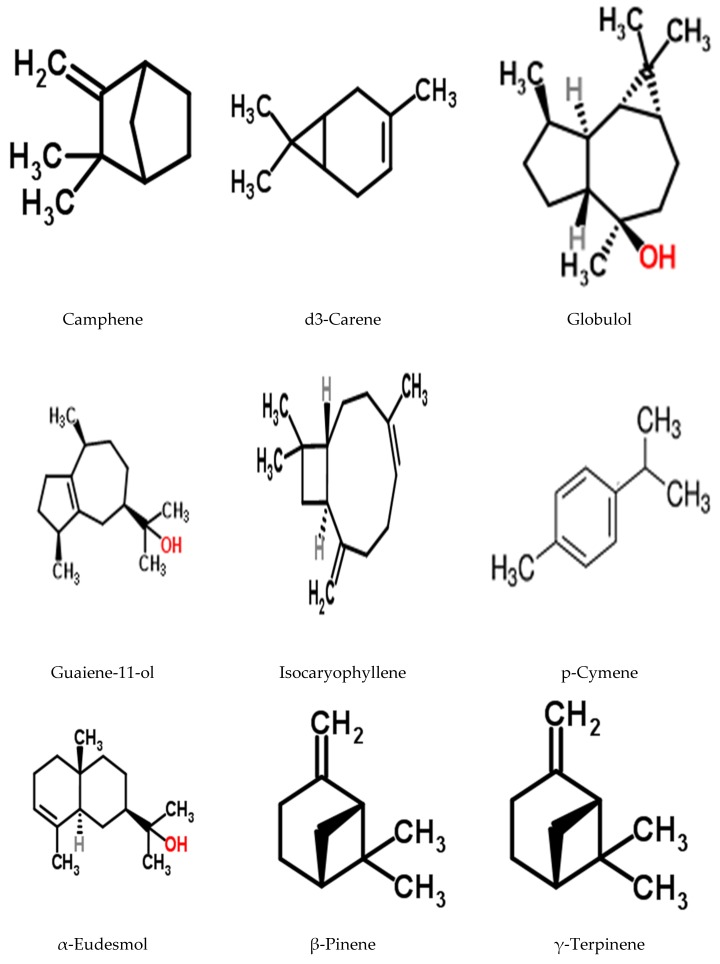
Some identified compounds from the root oil of *S. jollyanum.*

**Figure 4:**
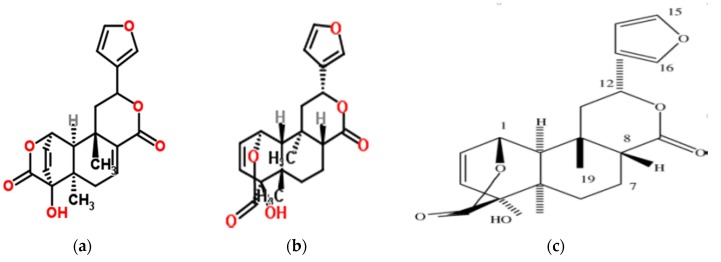
Isolated compounds from fruits of *S. jollyanum*. Structure of isolated fibleucin (**a**) and isocolumbin (**b**) [22], Structure of isolated columbine (**c**) [22].

The majority of the reported biological and pharmacological activity of *S. jollyanum* extracts (Table 2) has been attributed to their bioactive principles and constituent phytochemicals. A detailed phytochemical analysis of the ethanol root extract of *S. jollyanum* revealed that it contains compounds such as terpenoids and flavonoids, while alkaloids are reported to be the most dominant chemical constituents [19]. Phytochemical investigation by Nia et al. [4] revealed the presence of tannins, saponins, terpenes, and alkaloids in the different fractions of methanol extracts of the stem bark. The chloroform fraction was found to be the most active of all fractions, and it tested positive to the test of flavonoids and alkaloids. In the submission of Aboabaand Ekundayo [20], detailed analysis of the essential oil of *S. jollyanum* root using gas chromatography-mass spectrometry analysis (GC-MS) revealed a total of 19 compounds (α-pinene, α-ylangene, guaia-6,9-diene-4α-ol, globulol, guaiene-11-ol, α-eudesmol, isocaryophyllene, aromadendrene, selina-4(15),6-dien *E*-β-isocaryophyllene, γ-terpinene, γ-humulene, epi-zonarene, δ-amorphene, 1,8-cineol,camphene, B-pinene, p-cymene, d3-carene, Figure 3) consisting of monoterpenoids (33.5%) and sesquiterpenoids (56.3%), while (10.2%) of the total oil constituents remain unidentified. Phytochemistry of the seed extracts by Ibironke et al. [21] revealed the bio-availability of flavonoids, alkaloids, and saponins, while phylobatannin and free anthraquinone were not present. Proximate analysis of the seed extract showed the crude fat, moisture, crude protein, carbohydrate, ash, and fiber contents to be 9.65%, 16.70% 48.09%, 16.79%, 3.26%, and 5.51%, respectively, while the energy value was 1460 kcal/100 kg. This suggests that the fruits are an important source of nutrients and energy [21]. Interestingly, the flame photometric and atomic absorption spectrophotometric (AAS) analysis of the mineral element composition of the seeds showed an appreciable amount of macro and micro elements that are required for the growth in humans and animals [21]. Minerals such as calcium (8.92 mg/L), magnesium (0.44 mg/L), potassium (4.26 mg/L), iron (0.22 mg/L), manganese (0.19 mg/L), zinc (1.38 mg/L), and sodium (4.70 mg/L) were present in appreciable quantity. This indicates the importance of the plant in building strong bones, the production of energy, and carrying out some metabolic reactions in the body [21]. Previous scholarly articles documented the isolation of three furanoditerpenes (columbin, isocolumbine, and fibeucin) (Figure 4) and alkaloids (protoberberine) from the fruit [22]. 

## 5. Pharmacological and Biological Activities

### 5.1. Anti-Diabetic Activity

Investigation of the different extracts of morphological organs of *S. jollyanum* indicated its blood glucose lowering potential. The effect of petroleum ether seed extract on oral glucose tolerant test (OGTT) and alloxan-induced diabetic rabbits revealed that 1 g/kg of the extract administered 30 min before glucose load considerably reduced blood glycemic level by 20% relative to the untreated group. The study also reported the anti-hyperglycemic activity of the extract on alloxan-induced diabetic rabbits [29]. In another study, a 9-day treatment regimen revealed that the extract caused a significant (*p* < 0.05) dose-dependent decrease in the plasma glucose level from the 3rd to the 9th day. The three-dosage group showed a peak percentage decrease of 12.3%, 29.2%, and 32.7%, which compared favorably with glinbenclamide at 51.9%. Furthermore, the aqueous root extract demonstrated a dose-dependent reduction in blood glycemic level of alloxan-induced diabetic rabbits [30]. Ethanol extracts of *S. jollyanum* leaf at concentrations of 50, 100, 200 mg/kg significantly (*p* < 0.05) lowered the blood glycemic index of alloxan-induced diabetic rabbits in a dose-dependent manner, with plasma glucose level of 200.2 mg/100 mL (42.8%) at 200 mg/kg [31]. Alese et al. [32] reported that the methanol root extract demonstrated hypoglycemic effects on streptozotocin-induced diabetic Wistar rats. Oral dosage of 200 mg/kg extracts for 2 weeks caused a significant decrease in blood glucose to 6.62 mmol/L relatively to the uncontrolled group with blood glucose level of 16.3 mmol/L. The results of these studies validate the traditional claim of the blood glucose lowering activity of the plant, and thus may serve as a potential source of potent anti-diabetic compounds.

### 5.2. Antioxidant Activity

Studies on superoxide radical and hydrogen peroxide scavenging ability of the methanolic stem extract revealed a dose-dependent anti-oxidant activity with *IC*_50_ value of 13.11 μg/mL and 30.04 μg/mL when compared to ascorbic acid 15.34 μg/mL and 35.44 μg/mL respectively [23]. In a separate study, Olorunnisola and Afolayan[24] reported that the plant significantly (*p* < 0.05) ameliorated the oxidative stress related with *P. berghei* infection in mice, which was evident in the reduced levels of total protein and liver MDA (malondialdehyde). In addition, there was increased activity of serum and liver catalase (CAT), superoxide dismutase (SOD) and glutathione (GSH) levels. Alternatively, Nia et al. [4] point to the anti-oxidant activity of extracts of all morphological organs on 2,2-diphenyl-1-picrylhydrazyl hydrate (DPPH). The stem bark was observed to be the most active with *IC*_50_ of 1.80 μg/mL, when compared with ascorbic acid 0.80 μg/mL. The leaf had the weakest activity, *IC*_50_ of 4.35 μg/mL, while *IC*_50_ of the root bark was 3.50 μg/mL. Fractions of the stem bark were screened, and the chloroform fraction exhibited the most potent activity with an *IC*_50_ of 1.54 μg/mL. The findings emanating from these studies indicate the potential of *S. jollyanum* as an anti-oxidant (Table 2), and thus could be explored in the development of new pharmaceuticals. 

### 5.3. Anti-Inflammatory

Studies on the in vivo anti-inflammatory activity of *S. jollyanum* crude extracts and isolated compounds were investigated in healthy adult rats inoculated with carrageenan [22]. It was reported that the methanol fruit extract at a concentration of 200 mg/kg showed the stronger inhibition (79.58%) of oedema formation on hind paws, while root extract demonstrated a 53.75% inhibition. The same study also revealed that three furanoditerpenes (namely columbin, isocolumbine, and fibleucin) isolated from the methanolic fruit extract demonstrated considerable anti-inflammatory potentials. Columbin and flavonoids-rich fraction at 200 mg/kg exhibited 67.08% and 76.25% anti-inflammatory activities that were in a comparable range with acetylsalicylic acid. Olorunnisola et al. [27,28] evaluated the in vitro inflammatory potential and possible modes of action of crude extracts and secondary metabolites of *S. jollyanum* organs. The results also provided some vindication for the traditional usage of *S. jollyanum* in managing inflammatory-related diseases in the West African sub-region.

### 5.4. Anti-Allergy Activities

The anti-allergic study was performed on milk-induced leukocytosis and oesinophilia in mice. The ethanolic fruit extracts demonstrated a considerable dose-dependent decrease in the absolute eosinophil and lymphocyte counts. The results suggested the anti-allergy property of the fruit extract, and the mode of activity may involve multiple mechanisms due to phytochemical interactions [36]. 

### 5.5. Anti-Malarial Activities

Anti-malarial studies on the leaf and root extracts of *S. jollyanum* were reported by Olorunnisola in [24,34] respectively. The in vivo anti-plasmodial activity of methanol extracts was evaluated using chloroquine-resistant *Plasmodium berghei* NK67 strain-inoculated *S*wiss albino mice. The leaf and root extracts demonstrated statistically significant concentration-dependent activities of (74.4%) and (54.1%), respectively. However, the standard drug arthemether-lumefartrin had a better antimalaria activity (81.4%). Further research is necessary to identify and characterize the active components and determine the possible mode of activity.

### 5.6. Anti-Bacterial Activities

Aboaba and Ekundayo [20] studied the essential oil composition of the root extract of *S. jollyanum* against *Bacillus subtilis*, *Salmonellatyphi*, *Staphylococcus aureus*, *Bacillus cereus*, *Proteus mirabilis*, and *Pseudomonas aeruginosa.* It was observed that the essential oil was effective against *Bacillus subtilis* and *Pseudomonas aeruginosa* strains with inhibition zones of 10 mm and 9.0 mm, respectively, at 1000 μg/mL. In a separate study, Koleosho et al. [25] showed the plant extract to be a potent inhibitor of *S. typhi*. The moderate antimicrobial activity displayed supports the traditional use of the root as a laxative which aids proper bowel movements and digestion. 

### 5.7. Anti-Viral Activities

Moody et al. [37,38] revealed that the methanol extracts of the different morphological parts were assessed for their antiviral activities on polio virus Types 1, 2, and 3. It was observed from the study that the leaf and root extracts were active against polio virus Type 2. Additionally, hexane and methanol extracts were investigated and reported for their mosaic virus inhibitory potentials in cowpea. 

### 5.8. Haematological Activities

Methanol root and leaf extracts of *S. jollyanum* were investigated for possible hematopoietic activity in Wistar mice infected with chloroquine-resistant *Plasmodium berghei* NK67. Methanol extracts of leaf and root were administered orally for 7 days. The study revealed that there was a significant increase in the pack cell volume (PCV), mean corpuscular volume (MCV), mean corpuscular hemoglobin concentration (MCHC), and hemoglobin (Hb). There was also an observable elevation in red and white blood cell levels, with the exception of monocytes and neutrophils. The study is suggestive of the ability of the extract to stimulate hematopoietic stem cells [22,23,26].

### 5.9. Effect on Weight

The effect of leaf and root extracts on weight change was investigated in malaria and diabetic rat. It was observed that there was a significant (*p* < 0.05) increase in weight gain in Wistar mice treated with the extracts. Comparative analysis suggested that the extracts significantly prevented loss of weight in a concentration-dependent pattern in the extract-administered group when compared to the negative control [24,26,33,35]. Consequently, the physical status of the extract-treated animals was improved. This may be related to the ameliorative effect of the extracts to prevent the acute fluid loss, fat catabolism, and protein catabolism which are evident in weight loss. 

### 5.10. Hepatoprotective and Toxicological Studies

Scientific examination of the hepatoprotective potential of stem bark extract revealed that the extract significantly ameliorated/prevented liver damage in carbon tetrachloride (CCl_4_)-induced rats. The study showed that the extract considerably (*p* < 0.05) reversed the elevated aspartate aminotransferase (AST), alkaline phosphatase (ALP), alanine amino transferase (ALT), and total bilirubin, and decreased the level of total serum protein in a concentration-dependent pattern [23]. In the submission of Mbaka et al. [26,33], no mortality or morbidity was recorded in the 120-days administration of ethanol leaf extract during the toxicity studies. Detailed assessment also indicated that there was no obvious inflammation of the internal organs. In addition, there were no appreciable increases in serum AST and ALT in the extract-administered animals. This observation denotes that the extract poses no damaging effect on the liver. The result of the histological study on the liver tissue morphology confirmed that no toxic effects of the extract were visible. Furthermore, minimum cytotoxic dose (MCD50) of the methanol leaf extract was also investigated on Hep-2 (Human epithelia cell line) and it was found to range within 3.9 × 10^−3^ mg/mL [37]. The Ames microbial mutagenicity test of the root ethanol extract showed no statistically observable increase in the number of revertant colonies in the four strains of *S. typhimurium* *TA*_97_, *TA*_98_, *TA*_100_, and *TA*_102_ at any concentration. This indicates that *S. jollyanum* has no ability to cause mutation in relation to the in vitro assay [19]. According to Aboaba and Ekundayo [20], the toxicity of the essential oil of *S. jollyanum* to brine shrimp lethality test showed a lethal concentration *LC*_50_ of 84.87 ppm. Therefore, the observed safety level of the plant extracts vindicates its age-long ethno-pharmacological usage.

### 5.11. Hypolipidemic Activity

Effect of the ethanol root extract on serum lipid profile was investigated on streptozotocin-induced diabetic albino rats. It was noticed that there was no observable difference in total cholesterol (TC) amount of the extract- and glinbenclamide-administered animals. Additionally, there was a significant (*p* < 0.02) difference in antiartherogenic index (AAI) and high-density lipoprotein level in extract-treated group (0.77 ± 0.02 mmol/L) when compared to untreated infected group (0.85 ± 0.02 mmol/L) [32].

### 5.12. Antidepressant Activity

The anti-depressant effect of the ethanol extract of the root was evaluated on mice using forced swimming and tail suspension examination. The plant extract (100–1000 mg/kg) increased the duration of mobility in both models in a concentration-dependent manner. However, it was observed that the standard drugs imipramine and fluoxetine were 20–50 times more potent than the extracts. This implied that the antidepressant activity of the extract might be as a result of its modifying activity on monoamine transport and metabolism [39].

### 5.13. Anxiogenic Activity

Anxiogenic activity was carried out by administration of 100–1000 mg/kg of the ethanol extract. The animals exhibited anxiety-like effects dose-dependently in a similar way to those induced by caffeine (10–100 mg/kg), and this was in contrast to the anxiolytic effect of diazepam (0.1–1 mg/kg) [40,41]. The result validates the conventional use of the plant for its stimulatory effect on the central nervous system and as mood enhancer. 

### 5.14. Anti-Angiogenic Activity

Angiogenesis has been reported as a fundamental process in the transition of benign tumors to malignant ones, and therefore Nia et al. [4] evaluated the anti-angiogenic activities of the methanol extract of morphological organs using in vivo chick chorioallantoic membrane (CAM) angiogenesis assay. It was revealed that the stem bark had the most potent activity, with an *IC*_50_ value of 1.00 μg/mL. In addition, the chloroform fraction of the stem bark exhibited the strongest inhibitory *IC*_50_ (1.54 μg/mL) activity against the formation of new endothelial cells [4], thus validating the ethno-botanical usage of *S. jollyanum* as an important anti-tumor agent. 

### 5.15. Antipyretic and Analgesic Activities

Muko et al. [42] disclosed that the petroleum ether and methanol extracts of *S. jollyanum* leaves possess significant in vitro analgesic and antipyretic activities.

### 5.16. Reproductive and Sexual Activity

Investigation on the potential of the plant extracts to affect sexual activities and hormonal levels in laboratory animals was carried out by Owiredu et al. [43]. It was observed that there was an increased urge by the male animals to mount on the female for the first time, increased duration of ejaculation, and shortened refractory period. Furthermore, a decrease in post-ejaculatory, climbing and intromission latency was observed in the extract-treated animals. These are relevant perceptions of sexual performance and satisfaction. In addition, testosterone level was increased by four-fold and about there was a one-third increment in follicle stimulating hormone (FSH) activity. However, there was a contradictory report by Raji et al. [44] that pointed to a considerable reduction in the total sperm count, fertilization ability of the sperm cells, movement and swimming (asthenozoospermia) abilities. There was also a considerable increase in superoxide dismutase activity in relation to the testes and degeneration of seminiferous tubules. However, it was suggested that *S. jollyanum* could produce deleterious effects on reproductive ability, which can be measured as a function of poor sperm quantity (epididymal sperm count), quality (sperm movement, viability, structure), and gradual impairment, loss of function of the tissues and cells of the testes.

## 6. Conclusions

The importance of *S. jollyanum* in the traditional medicinal system of Africa cannot be over-emphasized. The medicinal value of the plant is owed to it richness in alkaloids, tannins, saponins, flavonoids, and essential oils. These bioactive principles have been reported to be responsible for the various pharmacological efficacies reported in this review. Hence, all morphological organs of the plant stand an important chance as a major source of potent therapeutic compounds useful in the management of several human diseases.

## Figures and Tables

**Table 1:** Taxonomical classification of *Sphenocentrum jollyanum* [5].

Kingdom	Plantae
Division	Magnoliophyta (Cronquist)
Subdivision	Magnoliophytina (Frohne and Jensen)
Class	Ranunculopsida (Brongn)
Subclass	Ranunculidae (Takht)
Suborder	Ranunculanae (Takht)
Order	Menispermales (Bromhead)
Family	Menispermaceae (Juss.)
Genus	Sphenocentrum (Pierre)
Species	Jollyanum

**Table 2:** Summary of pharmacological activities *of S. jollyanum.*

Pharmacological Activities	Part Used	Extracts	References
Angiogenic	Stem bark,	Methanol, Chloroform	[4]
Anti-Oxidant	Stem bark, Root, Leaf	Methanol, Hexane, Chloroform, Ethanol, Butanol, Aqueous	[4,23,24]
Anti-Bacteria	Root	Essential oil, Ethanol	[20,25]
Haematological	Leaf , Root	Methanol	[22,23,26]
Anti-Inflammatory	Fruit, Root,	Methanol, Ethanol, Aqueous	[22,27,28]
Anti-Diabetic	Root, Stem, Fruit, Leaf	Aqueous, Ethanol, Methanol	[29,30,31,32]
Hypolipidemic	Root	Ethanol	[32]
Hepatoprotective,Toxicological	Stem bark, Leaf, Root,	Methanol, Ethanol, Essential oil	[23,26,33]
Anti-Malaria	Leaf, Root	Methanol	[24,34]
Weight Loss Prevention	Leaf , Root, Seed	Ethanol, Essential oil	[24,26,33,35]
Anti-Allergy	Fruit	Ethanol	[36]
Anti-viral	Stem, Root, Leaf	Hexane, Methanol	[37,38]
Anti-depressant	Root	Ethanol	[39]
Anxiogenic	Root	Ethanol	[40,41]
Analgesic, Antipyretic	Leaf	Methanol	[42]
Sexual, Reproductive	Root	Ethanol, Methanol	[43,44]
